# Cost-Effectiveness of Prolonged Physical Activity on Prescription in Previously Non-Complying Patients: Impact of Physical Activity Mediators

**DOI:** 10.3390/ijerph20053801

**Published:** 2023-02-21

**Authors:** Linda Ryen, Stefan Lundqvist, Åsa Cider, Mats Börjesson, Maria E. H. Larsson, Lars Hagberg

**Affiliations:** 1 University Health Care Research Center, Faculty of Medicine and Health, Örebro University, 701 82 Örebro, Sweden; 2 Department of Health and Rehabilitation, Unit of Physiotherapy, Institute of Neuroscience and Physiology, Sahlgrenska Academy, University of Gothenburg, 405 30 Gothenburg, Sweden; 3 Center for Physical Activity Gothenburg, Region Västra Götaland, 413 45 Gothenburg, Sweden; 4 Department of Occupational Therapy and Physiotherapy, Sahlgrenska University Hospital/Östra, 416 85 Gothenburg, Sweden; 5 Center for Health and Performance (CHP), University of Gothenburg, 405 30 Gothenburg, Sweden; 6 Department of Molecular and Clinical Medicine, Institute of Medicine, Sahlgrenska Academy, University of Gothenburg, 405 30 Gothenburg, Sweden; 7 Department of MGA, Sahlgrenska University Hospital, Region Västra Götaland, 416 50 Gothenburg, Sweden; 8 Research, Education, Development and Innovation, Primary Health Care, Region Västra Götaland, 411 18 Gothenburg, Sweden; 9 Centre of Clinical Research and Education, Region Värmland, 651 82 Karlstad, Sweden

**Keywords:** cost-effectiveness, cost–utility, physical, prescriptions, health behavior, metabolic syndrome X, quality of life, physical therapy specialty, primary healthcare, mediating factors

## Abstract

In Sweden, physical activity on prescription (PAP) is used to support patients in increasing their levels of physical activity (PA). The role of healthcare professionals in supporting PA behavior change requires optimization in terms of knowledge, quality and organization. This study aims to evaluate the cost-effectiveness of support from a physiotherapist (PT) compared to continued PAP at a healthcare center (HCC) for patients who remained insufficiently active after 6-month PAP treatment at the HCC. The PT strategy was constituted by a higher follow-up frequency as well as by aerobic physical fitness tests. The analysis was based on an RCT with a three-year time horizon, including 190 patients aged 27–77 with metabolic risk factors. The cost per QALY for the PT strategy compared to the HCC strategy was USD 16,771 with a societal perspective (including individual PA expenses, production loss and time cost for exercise, as well as healthcare resource use) and USD 33,450 with a healthcare perspective (including only costs related to healthcare resource use). Assuming a willingness-to-pay of USD 57,000 for a QALY, the probability of cost-effectiveness for the PT strategy was 0.5 for the societal perspective and 0.6 for the healthcare perspective. Subgroup analyses on cost-effectiveness based on individual characteristics regarding enjoyment, expectations and confidence indicated potential in identifying cost-effective strategies based on mediating factors. However, this needs to be further explored. In conclusion, both PT and HCC interventions are similar from a cost-effectiveness perspective, indicating that both strategies are equally valuable in healthcare’s range of treatments.

## 1. Introduction

Globally, non-communicable diseases contribute to more than 70% of total deaths [1], with cardiovascular diseases as the most common cause of death and metabolic risk factors considered the most prominent for the global burden of disease [1,2]. Regular physical activity (PA) provides a basis for positive health effects, including the prevention and treatment of a plurality of non-communicable diseases [3,4,5]. However, only a minority of all adults reaches the internationally recommended PA level, including 150 min of moderate-intensity PA or 75 min of vigorous-intensity PA per week [6,7]. The economic burden of physical inactivity to societies around the world is substantial [8]. Although several PA interventions are considered cost-effective, there are factors complicating the interpretation of results in published research, such as short time perspectives, the measurement of single treatment effects only, the variability of interventions in different population groups and a lack of cost estimates and savings in the cost-effectiveness analyses [9,10,11].

The physical activity on prescription (PAP) method used in Swedish healthcare by licensed healthcare professionals includes patient-centered counselling, individualized PA recommendations with a written prescription and individualized structured follow-ups. From the patient’s perspective, it seems crucial to individualize all parts of the PAP treatment in order to reinforce behavior changes towards increased PA [12,13,14]. A systematic review of Swedish PAP found a high level of evidence that physically inactive patients in the healthcare setting increased their PA levels [15,16].

Previous studies of PAP have evaluated the effects of shorter interventions, but do not provide guidance on how healthcare providers should act when patients do not reach sufficient levels of PA within this time frame. Hence, there is a need for further studies on long-term PAP interventions with longer follow-up periods [17,18]. Lifestyle change is usually an ongoing process that takes several years [19] and is affected by mediating factors associated with increased PA, such as enjoyment, outcome expectations and confidence in succeeding in changing the PA level [20,21,22]. These factors, defined as intervening causal variables, are important in creating a cause–effect pathway between an intervention and PA [23,24] and could optimally be part of the patient-centered work of tailoring interventions with different levels of support. Immediate rewards of PA (e.g., enjoyment) predict long-term adherence to the PA, whereas delayed rewards (e.g., health benefits) do not [25]. Therefore, it is likely that those who experience high enjoyment do not need any support at all to adhere to PA, and the lower the experienced enjoyment is, the greater is the need of support for sustainable PA. Outcome expectations represent the belief that a behavior change, e.g., increased PA, will lead to a certain outcome [26]. Although not consistent across studies, outcome expectations are considered important in predicting PA behavior [27,28]. Confidence, or self-efficacy expectations, is described as the confidence in one’s capability to change one’s behavior (e.g., PA) [29]. Having confidence in the readiness to change the PA level has been shown to be strongly associated with PA [30]. In this study, the mediating factors of enjoyment, outcome expectations and confidence were measured and have been described in detail previously [31,32]. As behavior changes take time [19], the question is how healthcare providers should act when the desired effect on PA levels is not achieved after a certain period of time, even though the patient is motivated to continue with PAP. As far as we know, there are no previous studies showing what healthcare should do when a lifestyle intervention has failed, which, according to the literature, is a common situation [17,33,34].

Economic evaluations of health interventions compare the costs and consequences of different strategies in order to provide decision-makers with information regarding choices affecting health and the use of resources. Traditionally, these analyses provide answers as to which method is most cost-effective for the average patient. However, recently updated international guidelines on the reporting of health economic evaluation results, known as the Consolidating Health Economic Evaluation Standards or CHEERS statement, include new recommendations on subgroup analyses, acknowledging that heterogeneity among patients means that strategies might be cost-effective for specific groups while not for others [35].

The main aim of this study is to evaluate the cost-effectiveness of a three-year prolonged program of enhanced PAP support delivered by a physiotherapist (PT) compared to continued (standard) PAP treatment at the healthcare center (HCC) for patients who remained insufficiently physically active after a prior six-month period of PAP treatment in a primary healthcare setting. A secondary aim was to explore whether enjoyment, expectations and confidence have potential in identifying cost-effective strategies on a subgroup level.

## 2. Materials and Methods

### 2.1. Study Design and Study Population

This cost–utility analysis was based on a randomized controlled trial (RCT) [31] of PAP treatment conducted with two intervention arms: one PT group and one HCC group. The time horizon was three years, and the analysis was performed from both a healthcare and a societal perspective. The study was approved by the Regional Ethical Review Board in Gothenburg, Sweden (ref: 529-09).

The present analysis forms part of a long-term follow-up study including 444 patients, which has been described previously [32,36]. Out of these patients, 190 patients did not achieve the internationally recommended minimum PA level after six months of PAP treatment, and were thus included in this study. These 190 patients were living in an urban area of Gothenburg, Sweden. The patients were 27–77 years of age, and had at least one metabolic risk factor (Table 1). Before inclusion in the study, they received standard PAP treatment for six months during 2010–2014 at one of 15 designated healthcare centers in Gothenburg. At the six-month follow-up, 56% of the 190 patients had increased their PA level to some extent, but none of the included patients reached a sufficiently high PA level according to the internationally recommended minimum of 150 min/week. PA level was assessed via two questions regarding moderate- and vigorous-intensity PA during the past week. The patients agreed, orally and in writing, to participate in the RCT at the six-month follow-up, and were then randomized to either enhanced PAP treatment provided by a physiotherapist (PT group, n = 98) or continued ordinary PAP treatment delivered by nurses at the healthcare center (HCC group, n = 92). Randomization was based on block randomization, with an automated computer-based stratification of age, sex and BMI. Each patient was then contacted by the PT or HCC for further intervention. A more detailed description of the study population has been published previously [31].

### 2.2. Intervention

The PA and PAP interventions were offered to the patients according to the *Physical Activity in the Prevention and Treatment of Disease* (FYSS) handbook and the concept of the Swedish PAP model [37,38]. The intervention is described in detail elsewhere [31].

In the HCC group, PAP treatment was provided by nurses, whose area of expertise was nursing, who were trained on the health effects of PA and on treatment with PAP. The treatment included an individualized dialogue concerning PA, an individually dosed PA recommendation including a written prescription and an individually adjusted follow-up. The majority of the patients received continued PAP treatment at follow-ups 2–3 times a year during the intervention period.

The physiotherapists, whose area of expertise was work physiology, who provided treatment in the PT group, were also educated in PAP treatment. The PT intervention included the first two individually adapted parts described for the HCC group—that is, the individualized dialogue and the individual PA recommendation. The third part (the follow-up) differed between the two interventions, and in the PT group, this was arranged via a fixed follow-up schedule. This schedule contained a total of ten follow-up sessions during the three-year intervention: six during the first year of intervention, three during the second year and the final one at the three-year follow-up. The PT group also received five additional aerobic physical fitness tests during the intervention period, using an ergometer bicycle. The results from the physical fitness test formed the basis for a continuing dialogue with the patient concerning PA and for an individual dosage of PA regarding frequency, duration and intensity, recorded in a written prescription.

### 2.3. Measurements

The patients’ own costs, health-related quality of life (HRQOL), healthcare resource use and absence from work were measured at baseline and at the one-, two- and three-year follow-ups. Costs were estimated based on data from the follow-up questionnaires and administrative sources, as described below for each type of cost included. Unit prices used for estimation are summarized in Table 2. Costs were expressed as 2018 prices and a yearly discount rate of 3% was applied. HRQOL was measured by the Swedish version of the Short Form 36 (SF-36 Standard Swedish Version 1.0) [39], transformed to quality-adjusted life years (QALYs) with SF-6D [40] and a UK tariff [41]. The UK tariff is the one commonly applied also to a Swedish population as there are no Swedish tariffs available. Details on the HRQOL in both groups at each time point are available in Supplemental Table S1.

Estimations of the cost-effectiveness are presented both from a healthcare perspective and from a societal perspective. The healthcare perspective includes the healthcare resource use in terms of intervention costs as well as costs for visits to primary care or hospital. For the societal perspective, of which healthcare resource use forms a part, individual expenses for PA, production loss due to sick leave and the time cost of exercise are added.

The amount of healthcare resource use in outpatient care was based on the self-reported number of visits to primary healthcare centers and hospitals stated in the yearly follow-up questionnaires. The number of visits to the physiotherapist in the PT group was reported from the administrative source in the study. The costs for all healthcare resource use were estimated based on unit costs differentiated by professions according to standard production prices negotiated for the trade of healthcare between county councils [42] and stated in 2018 prices.

Individual expenses related to PA, such as the costs of equipment or transportation, were reported by the patients in the yearly follow-ups. Patients stated their expenses for the last month, which were then multiplied by 12 to estimate yearly expenses. Since different individuals had entered the interventions in different years, all expenses were converted to 2018 prices using the Swedish consumer price index (CPI). Conversion to USD was based on the mean exchange rate on 1 January 2018 (1 USD = 8.78 SEK).

The cost of increased exercise time was estimated based on the experience of exercise time in comparison to the experience of leisure activity forgone and of household work [43]. The mean net salary in Sweden was used in the estimation [44]. Time spent on exercise was measured with the International Physical Activity Questionnaires [45]. When experience of PA time was rated higher than leisure activity forgone, there was no time cost. When experience of PA was rated lower than household work (cleaning), the time cost was set to the same as for half net salary. When experience of PA was rated in between the experience of household work and that of leisure activity forgone, the cost was set to the part of the half net work salary that corresponded to the relative position between experience of household work and leisure activity forgone.

Individuals were asked about the amount of sick leave from paid work in the yearly follow-ups, and their answers were then converted to full days of absence from work. Each full day of sick leave was then valued in accordance to the human capital approach, based on average wages including payroll taxes [42,46]. Production loss was only estimated for those who stated that they were absent from paid work.

### 2.4. Mediating Factors for Increased PA

Based on the positive relationship between PA and health, mediators for increased PA can also be seen as mediators for improved health. Enjoyment was measured using the Physical Activity Enjoyment Scale (PACES) [47], modified by Motl et al. [48], including 16 positively or negatively worded items rated on a 5-point Likert scale (1: Does not apply at all, 5: Truly applies). The negatively worded items were reverse-scored, and the responses were added to a score that ranged from 16 to 80.

Outcome expectations were assessed with the Outcome Expectations for Exercise-2 Scale (OEE-2) [49,50], including 13 positively or negatively worded items also rated on a 5-point Likert scale (1: Strongly agree, 2: Strongly disagree). The negative OEE items were reverse-scored, and the numerical ratings for each response were summarized and divided by the number of items where a highly valued outcome expectation from the patient gave a low total score.

Confidence (the readiness to change PA level) was measured via a 100-mm visual analogue scale (VAS) with the question “How confident are you about succeeding with changing PA level?” [51,52]. The VAS line was anchored at each end with words that described the minimum (not at all) and maximum (very) extremes. The mediating factors have been described in detail previously [32].

### 2.5. Health Economic Analysis Methods

In a cost–utility analysis, i.e., a cost-effectiveness analysis with QALY as the outcome measure, costs and effects for at least two alternative treatments are compared in terms of their costs and effects, resulting in an incremental cost-effectiveness ratio (ICER). Here, the costs included for each treatment were actual healthcare resource use, intervention costs, individual expenses related to PA, estimations of production loss due to work absence and individualized time cost for PA. The effect was measured in terms of changes in HRQOL expressed as quality-adjusted life years (QALYs).

The cost-effectiveness of the PT group compared to the HCC group is presented in terms of the incremental cost-effectiveness ratio (ICER), which represents the cost of achieving one additional QALY when applying PAP supported by PT compared with continued PAP by HCC. This is expressed by

$$ICER=\frac{{Cost}_{PT\;group}-{Cost}_{HCC\;group}}{{QALY}_{PT\;group}-{QALY}_{HCC\;group}}$$

To include the mediating factors in the analysis, patients were divided into two subgroups for each factor: the half who, at the start of the study, experienced the lowest versus the highest enjoyment, outcome expectations and confidence, respectively, according to the median value in each of the measurements. ICERs were then estimated comparing the costs and effects of the PT intervention compared to HCC treatment for all subgroups, respectively, following the below example for the patients reporting high enjoyment (≥58). The corresponding ICERs were then estimated for low enjoyment (<58), high confidence (≥55), low confidence (<55), high expectations (<2.08) and low expectations (≥2.08).

$$ICER=\frac{{Cost}_{PT\;high\;enjoyment}-{Cost}_{HCC\;high\;enjoyment}}{{QALY}_{PT\;high\;enjoyment}-{QALYs}_{HCC\;high\;enjoyment}}$$

**Figure 1:**
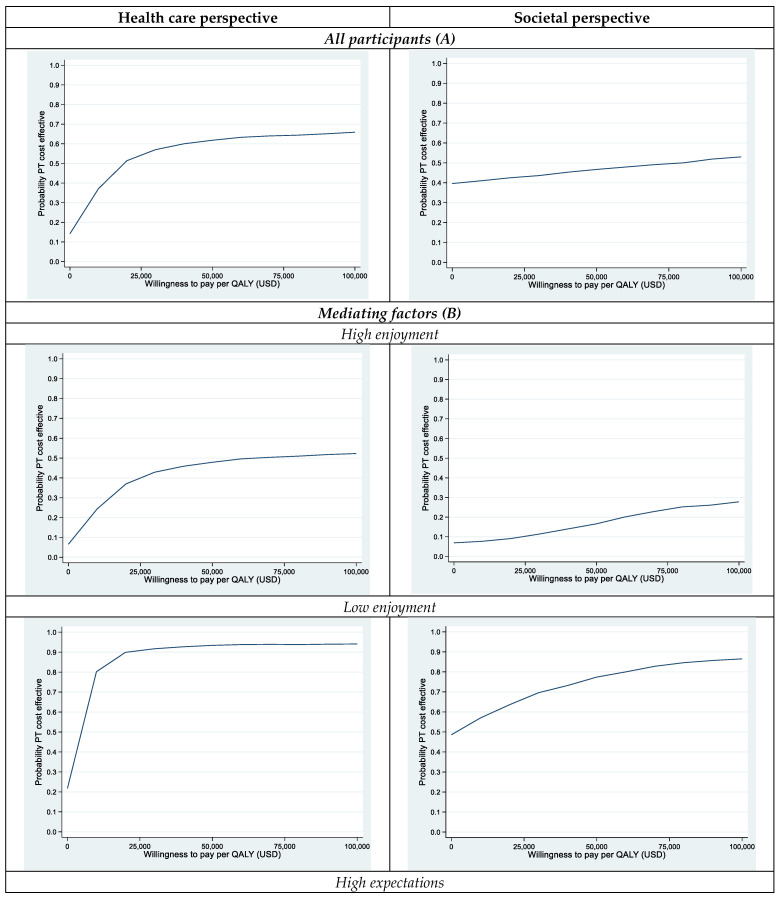

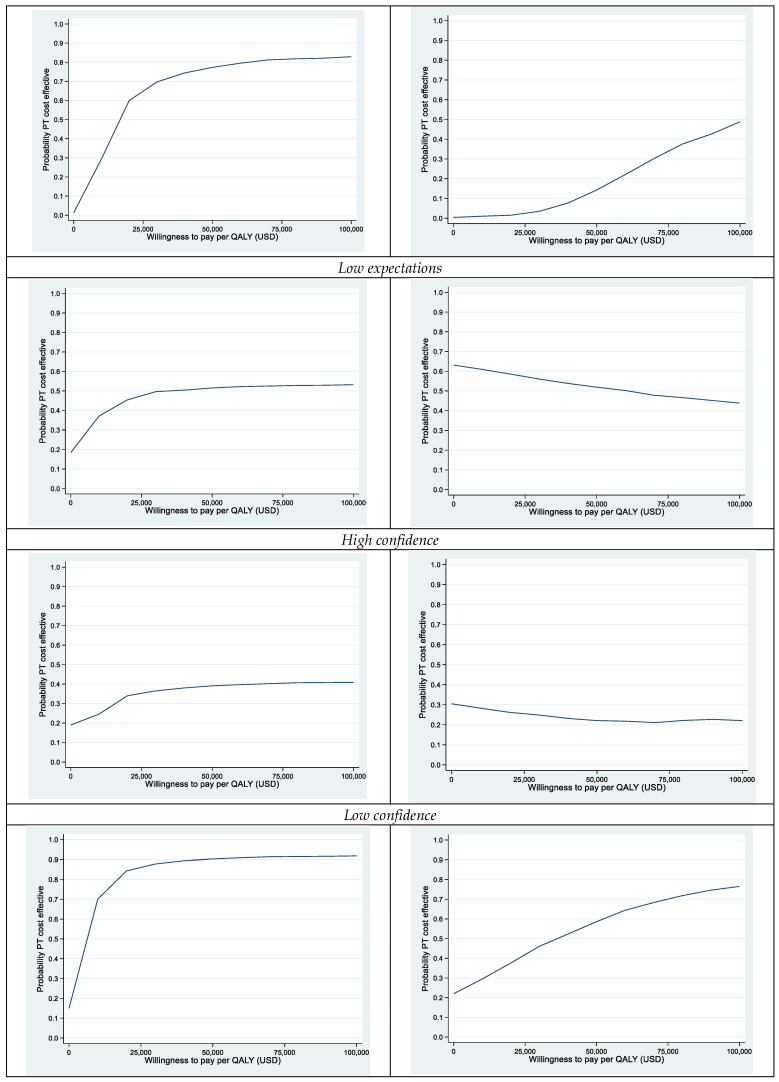
Cost-effectiveness acceptability curves for (**A**) all participants and (**B**) subgroups based on mediating factors.

Bootstrapping was performed to acknowledge uncertainty in both costs and effects. This procedure takes the variance in the trial data into account by repeatedly drawing random samples (of the same size as the original) with replacements of costs and effects from the two groups. In this case, 1000 new samples were drawn. Using the net monetary benefit method, QALYs are then replaced by varying willingness-to-pay (WTP) levels for gaining a QALY, in this case ranging from USD 0 to USD 1,000,000. The results of this analysis are presented as cost-effectiveness acceptability curves (CEACs) (Figure 1) showing the probabilities for the PT treatment to be the most cost-effective choice at different WTP thresholds [53]. When the curve is above the 0.5 line (on the vertical axis), this means that PT is more likely than HCC to be the most cost-effective choice for the WTP on the horizontal axis.

All randomized participants were kept in their original study groups. For missing data needed to estimate costs and effects, stochastic imputation (by using a single dataset from multiple imputation) was performed based on the assumption that data were missing at random. All analysis was performed on the imputed dataset.

For the subgroup analyses on mediating factors, only complete cases on each mediator, respectively, were used, and patients with costs more than three standard deviations from the mean were excluded.

## 3. Results

At the three-year follow-up, 70% of the patients in the PT group (n = 69) and 66% of the patients in the HCC group (n = 61) attended. Of the patients attending the follow-up, 77% (*p* < 0.001) of the PT group (n = 61) and 66.1% (*p* < 0.001) of the HCC group (n = 59) had increased their PA level and 44.3% vs. 35.6% had achieved the public health recommendation of ≥150 min of moderate-intensity PA per week. There were no significant differences in PA level between the groups at the three-year follow-up (*p* = 0.55).

In the PT group, the incremental QALY gain per participant compared to the HCC group over three years was 0.016, see Table 3 below. From the societal perspective, the average cost per participant amounted to USD 13,488 in the PT group and USD 13,219 in the HCC group. From the healthcare perspective, the corresponding costs were USD 2685 in the PT group and USD 2150 in the HCC group. According to these costs and effects, the resulting ICER was USD 16,771 per additional QALY gained from the societal perspective and USD 33,450 per additional QALY gained from the healthcare perspective for the PT group compared to the HCC group.

Based on bootstrapping, taking the variability in the sample into consideration, cost-effectiveness acceptability curves (CEAC) were produced (Figure 1). In order for PT to be more likely than HCC to be cost-effective for the whole sample, the willingness to pay for a QALY needed to be higher than USD 57,000 when considering the societal perspective and higher than USD 22,000 when considering the healthcare perspective (Figure 1). This can be related to a willingness to pay of USD 57,000 for a QALY (corresponding to SEK 500,000, a threshold value commonly referred to in Sweden). Cost effectiveness scatterplots for the CEACs are available in Supplemental Figure S1.

In a second step, after splitting the sample into high/low on the mediating factors enjoyment, outcome expectations and confidence, CEACs were produced for these subgroups as well.

## 4. Discussion

### 4.1. Main Outcomes

The main aim of this study was to evaluate the cost-effectiveness of a three-year prolonged program of enhanced PAP support delivered by a physiotherapist compared to continued (standard) PAP treatment at the healthcare center for patients who remained insufficiently physically active after a prior six-month period of PAP treatment in a primary healthcare setting. We have tried to shed light on what healthcare should do when a short-term lifestyle intervention is not enough for patients to achieve a desirable PA level. This study does not allow for the analysis of whether the patients “got their chance” and nothing more should be done, but we highlight whether it is most cost-effective to continue the intervention started (HCC group) or to enhance it (PT group). The cost per QALY for the PT strategy compared to the HCC strategy was USD 16,771 with a societal perspective and USD 33,450 with a healthcare perspective. Given a willingness to pay of USD 57,000 for a QALY, the probability of cost-effectiveness for the PT strategy compared to the HCC strategy was 0.5 with a societal perspective and 0.6 with a healthcare perspective.

There are no formally established thresholds, but cost-effectiveness ratios of 50,000–100,000 USD in the USA and 32,000–50,000 USD in the UK have often been accepted [54]. The World Health Organization argues that a threshold should simply be seen as an indication of poor, good or very good value for money [55]. There are no general recommendations for the threshold for the probability of cost-effectiveness for a change in routine care, but there are arguments that it should be close to 0.50 [56]. Consequently, it can be concluded that base-case results indicate that PT is cost-effective compared to HCC, but the uncertainty is large. Therefore, it was not possible to draw a definite conclusion about the most cost-effective PAP strategy in this study, as neither of the strategies was clearly superior to the other. The subgroup analyses showed that when enjoyment was high, the HCC intervention was most cost-effective, and when enjoyment was low, the PT intervention was the preferred choice. For confidence and expectations, the result was ambiguous, with small differences or different results depending on perspective. The number of participants in each subgroup was small and the result should be seen as an indicator of the possible impact of mediators. Nevertheless, the analysis showed that it may be worth considering the individual patient’s mediators for increased PA before agreeing with the patient on choosing the optimal intervention. It is probably advantageous to be able to offer either of the methods for increased individualization of the support, where the patient’s preferences are integrated as a vital part of evidence-based medicine [57].

In this study, the two interventions were quite similar in terms of cost-effectiveness. At the same time, the subgroup analyses indicated that they were not equal in effect and cost-effectiveness for everyone. In particular, the subgroup analysis based on enjoyment showed different cost-effectiveness for the respective interventions. Enjoyment has been shown to be the most important mediator for increased physical activity [58,59] and consequently the degree of enjoyment could affect whether extensive support is needed for the individual. In this study, individuals were randomized to either the PT or HCC group. This study suggests that as some individuals seem to benefit more from increased support, cost-effectiveness might be enhanced by screening for enjoyment together with other individual preferences. In clinical use, before the decision about which type of intervention to choose, screening of enjoyment could easily be performed with the PACES short version [60], with four questions instead of 16, as in this study.

As the subgroup analysis indicated, the HCC intervention was more cost-effective for patients with higher enjoyment. For patients with lower enjoyment, the PT intervention seemed to be more cost-effective. However, the PT intervention was problematic to implement, with relatively low compliance (with an attendance rate of 5.8 out of 11 follow-ups). Possible explanations could be a lack of time, transport problems or a lack of motivation. It is therefore important that only patients who have the need and motivation for this type of support are offered it. More knowledge is needed on whether the areas of expertise of different professional groups (nurses—nursing, physiotherapists—work physiology), in addition to training in PA and PAP, have significance for the patient’s opportunity to increase their PA.

### 4.2. Strengths and Weaknesses

It is not obvious how healthcare professionals should treat patients who have begun the process of changing their PA level but not succeeded in achieving the desired result in the short term. The patients in this study were motivated to continue the process of behavior change, and so we did not consider it ethically acceptable to randomize some of the patients to discontinued treatment—that is, to a “do nothing” group. This means that the analysis was limited to the way in which continued support should be provided, and not to the question of whether it is cost-effective to continue to provide support or not.

The study was carried out in a real-world setting, which makes the results generalizable. It also means that the study could not be carried out completely according to protocol. The inclusion of the patients in the study took much longer than planned, and the interventions were implemented on the basis of each patient’s condition and motivation. This resulted in an average of 5.8 PT counselling sessions instead of the planned 11 sessions, indicating that the patient group as a whole was not motivated to participate in such an extensive intervention.

As in all long-lasting interventions, there was an increase in missing data over time. This was handled by multiple imputation (but using one dataset instead of a large number of datasets, which is otherwise usual, since the calculation of the probability of cost-effectiveness requires a single outcome at the individual level) based on the assumption that data were missing at random. This could have led to biased results if those who were least physically active were those who had the most dropouts. However, there is no reason to believe that this would have differed between the two groups.

The groups differed in terms of the initial QALY level, which might be a concern since a lower level means greater potential for improvement. However, the difference was due to randomization and not to systematic factors.

As there are no specific Swedish tariffs available, preference weights for estimating QALYs are based on UK tariffs. This is standard procedure when using the SF6D in Sweden.

Many cost-effectiveness analyses of PA interventions have considered the time cost of exercise, but all of them were based on assumptions. As far as we know, this is the first attempt to base exercise costs on empirical data.

The study concerns two questions that are rarely answered in the research. Should healthcare continue to support a lifestyle intervention when it is not enough for patients to achieve a desirable PA level in the short term? The question cannot be answered based on our design, but we can see, after the first six-month period, a continued increase in PA, health and HRQOL among the patients at a relatively low cost [32]. This suggests that it can be cost-effective to prolong the intervention. The second question is whether healthcare should start with a small intervention and then increase in magnitude or invest in the most effective (and probably most expensive) intervention directly? This question also cannot be answered with certainty. However, 60% of the original patient group needed, at least temporarily, only a small intervention [36]. The remaining 40%, who were not helped during the first 6-month period, had improved PA, health and HRQOL with prolonged intervention, suggesting that an individualized step-by-step increase may be the best use of resources in this case.

### 4.3. Strengths and Weaknesses in Relation to Other Research

As far as we know, this is the first study of the cost-effectiveness of prolonged PAP or other prolonged PA interventions. However, earlier studies indicate that it is effective and thus probably also cost-effective to support change over a long period of time [19]. It has been shown that behavior change processes and the establishment of new PA habits are individual and, in many cases, take a long time, often several years. To increase the understanding of the behavior change process and promote behavior change maintenance in PA, more frequent measurements of mediators and outcomes are needed at longer time points [61]. Marcus et al. [19] recommend that follow-up should take place for at least 2 years, preferably 5–10 years.

### 4.4. Future Research

Lifestyle interventions rarely succeed for all patients, and there is very little research into how healthcare providers should act in cases where they fail to support the patient´s behavior change. This study and health economic analysis is one of very few attempts to shed light on the matter. There is a great need for more research in this important area about prolonged physical activity support in previously non-complying patients. Our study represents only an initial contribution. We believe that the following questions are important to highlight in the continued research in effectiveness as well as cost-effectiveness analysis:

(1) How long and to what extent should prolonged support be provided to non-complying patients?
(2) How should the prolonged support be organized?
(3) Are there individual factors in addition to enjoyment that can be the basis for individualizing the prolonged support?

## 5. Conclusions

Both PT and HCC interventions are quite similar from a cost-effectiveness perspective, indicating that both PAP strategies seem to be equally valuable to have in healthcare’s range of treatments. Individual preconditions for being physically active vary and so does the need, concerning time and magnitude, for professional support.

## Figures and Tables

**Table 1:** Baseline characteristics of the patients in the physiotherapist (PT) and healthcare center (HCC) groups.

Variable	PT Group	HCC Group	*p* Value
**Age ^a^ in years**	56.4 (10.2)	57.5 (11.3)	0.466 ^c^
**Sex ^b^**			0.888 ^d^
Female	48 (49.0)	46 (50.0)	
Male	50 (51.0)	46 (50.0)	
**Social situation ^b^**			0.082 ^d^
Single	44 (46.3)	30 (34.1)	
Married/cohabiting	47 (49.5)	52 (59.1)	
Other	4 (4.2)	6 (6.8)	
**Economic status ^b^—perceived**			0.594 ^d^
Good	56 (57.7)	54 (60.7)	
Neither nor	26 (26.8)	20 (22.5)	
Bad	15 (15.5)	15 (16.9)	
**Education ^b^**			0.829 ^d^
Elementary grade	16 (16.5)	19 (21.3)	
Upper secondary school	37 (38.1)	25 (28.1)	
University college	44 (45.4)	45 (50.6)	
**Tobacco ^b^**			0.532 ^d^
Smokers	9 (9.4)	12 (13.5)	
Non-smokers	64 (66.7)	57 (64.0)	
Ex-smokers	23 (24.0)	20 (22.5)	
**Diseases ^b^**			
Overweight/obesity	88 (89.8)	79 (88.8)	0.820 ^d^
Hyperglycemia	35 (36.5)	33 (37.1)	0.931 ^d^
Hypertension	75 (77.3)	75 (83.3)	0.304 ^d^
Hyperlipidemia	50 (52.1)	56 (62.2)	0.164 ^d^
Depression	16 (16.3)	8 (9.0)	0.135 ^d^
Musculoskeletal disorders	15 (15.3)	10 (10.9)	0.415 ^d^
Other	52 (53.1)	34 (38.2)	0.042 ^d^

Data are given as ^a^ mean (standard deviation), and as ^b^ number (percentage). Difference between PT and HCC group; *p* value was determined by ^c^ independent-samples *t*-test or by ^d^ Mann–Whitney U-test. Statistical significance was set at *p* ≤ 0.05.

**Table 2:** Input data for cost estimations.

Variable		Reference/Comment
*Healthcare resource use*		
Cost per PT visit	USD 74	Standardized prices for debiting out-of- county patient costs [42]
Average (actual) number of PT visits	5.8	From the study
Primary care visit		
Doctor	USD 182	Standardized prices for debiting out-of-county patient costs [42]
Other (including PT)	USD 74	Standardized prices for debiting out-of-county patient costs [42]
Hospital visit		
Doctor	USD 417	Standardized prices for debiting out-of-county patient costs [42]
Other	USD 179	Standardized prices for debiting out-of-county patient costs [42]
*Societal costs*		
Cost per day of sick leave	USD 170	Statistics Sweden
Time cost of exercise	Individual	Hagberg et al. [43]
Discount rate (per year)	3%	Standard discount rate

**Table 3:** Average effects and costs (standard errors) in the PT and HCC groups.

Variable	PT Group	HCC Group
QALY change	0.043 (0.023)	0.027(0.026)
Healthcare resource use	USD 2259	USD 2150
Intervention cost	USD 426	--
*Sum of costs healthcare perspective*	*USD 2685 (187)*	*USD 2150 (230)*
Individual expenses	USD 963	USD 1123
Production loss	USD 9679	USD 9679
Time cost of exercise	USD 160	USD 267
*Sum of costs societal perspective (incl. healthcare)*	*USD 13,488 (2774)*	*USD 13,219 (3154)*

PT = physiotherapist, HCC = healthcare center, QALY = quality-adjusted life year.

## Data Availability

The data presented in this study are available on request from the corresponding author.

## Supplementary Materials

The following supporting information can be downloaded at: https://www.mdpi.com/article/10.3390/ijerph20053801/s1, Figure S1: Cost-effectiveness scatterplots based on bootstrapping. PT vs HCC group; Table S1: Characteristics in health related quality of life in the physiotherapist (PT) and healthcare center (HCC) groups at each time point.

## Trial Registration

ClinicalTrials.gov ID: NCT03012516.
