# Supplementary material for: Cost-Effectiveness of Prolonged Physical Activity on Prescription in Previously Non-Complying Patients: Impact of Physical Activity Mediators
Source: Int J Environ Res Public Health. 2023 Feb 21;20(5):3801. doi: 10.3390/ijerph20053801 (PMC10001088; doi:10.3390/ijerph20053801)

Supplementary Table S1. Characteristics in health related quality of life in the physiotherapist (PT) and healthcare center (HCC) groups at each time point.

Variable[a]	PT group (n=98)				HCC group (n=92)			
	BL	1-year	2-year	3-year	BL	1-year	2-year	3-year
HRQOL SF-36, score:								
Physical functioning	79.2 (22.8)	76.7 (22.8)	77.9 (22.4)	79.0 (21.3)	79.6 (19.4)	82.9 (18.1)	79.7 (19.6)	78.2 (20.6)
Role limitation, physical	75.3 (34.9)	66.3 (41.8)	67.9 (39.9)	77.0 (34.9)	74.7 (36.1)	82.2 (32.4)	72.3 (37.4)	78.6 (35.5)
Bodily pain	67.4 (28.5)	66.8 (29.1)	64.0 (29.2)	66.9 (29.2)	66.4 (27.8)	72.1 (26.4)	65.1 (26.2)	64.4 (23.9)
General health	59.2 (21.9)	58.6 (22.1)	59.2 (22.5)	58.1 (21.1)	62.1 (20.4)	63.7 (20.3)	61.7 (19.7)	59.4 (19.8)
Vitality	51.4 (21.5)	55.8 (21.7)	52.5 (24.6)	53.2 (23.6)	55.0 (21.3)	60.2 (20.8)	55.2 (19.8)	57.4 (19.9)
Social function	78.6 (24.1)	80.5 (25.0)	80.1 (25.9)	81.8 (23.2)	82.4 (22.5)	85.3 (34.1)	80.8 (24.3)	84.9 (21.4)
Role limitation, emotional	68.8 (41.6)	74.9 (37.9)	74.4 (38.8)	77.0 (36.0)	77.9 (36.4)	81.8 (34.1)	74.9 (36.3)	81.2 (35.0)
Mental health	69.2 (20.0)	72.8 (19.4)	70.7 (20.1)	71.2 (19.0)	71.9 (19.6)	73.3 (20.5)	69.9 (21.3)	74.0 (20.4)
Physical component summary	46.6 (11.3)	44.0 (12.1)	44.2 (12.9)	45.4 (11.6)	45.6 (10.2)	47.9 (8.7)	45.6 (10.0)	45.5 (9.1)
Mental component summary	42.7 (13.3)	46.2 (12.3)	45.0 (13.0)	45.1 (11.4)	46.1 (11.4)	46.9 (10.7)	44.7 (12.3)	47.7 (12.1)

BL, baseline; HRQOL SF-36, Health Related Quality of Life 36-Item Short Form Health Survey.

Data are given as [a] *mean (standard deviation).*

Supplementary Figure S1. Cost-effectiveness scatterplots based on bootstrapping. PT vs HCC group

a) Societal perspective

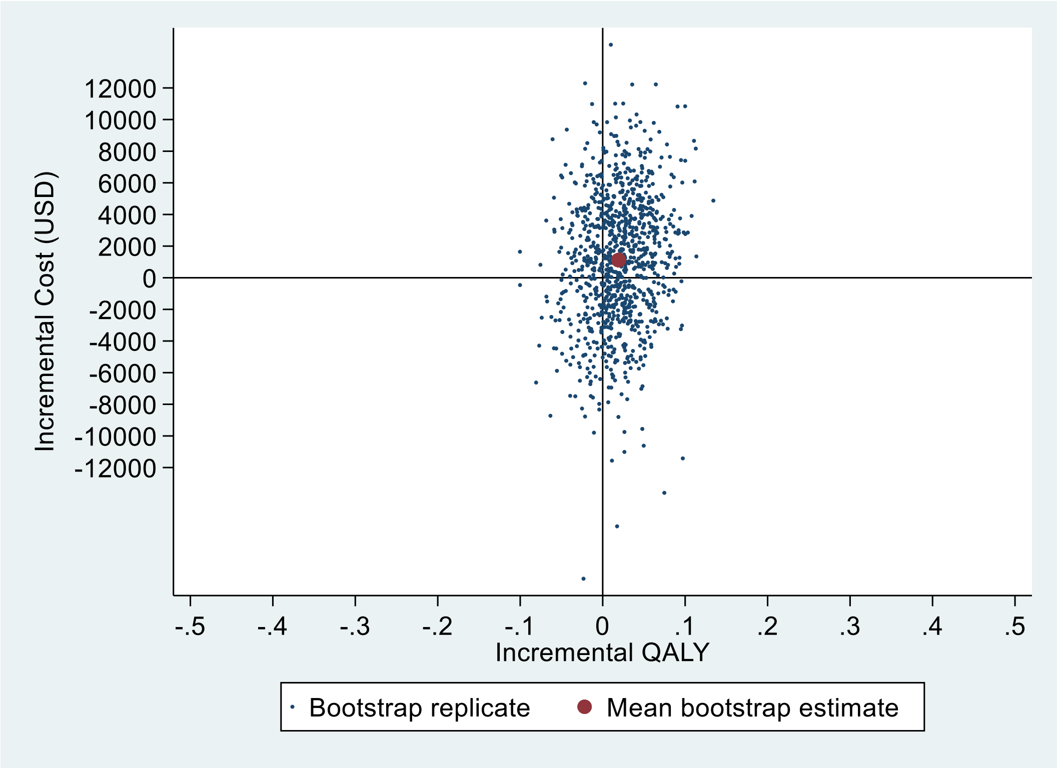

b) Health care perspective

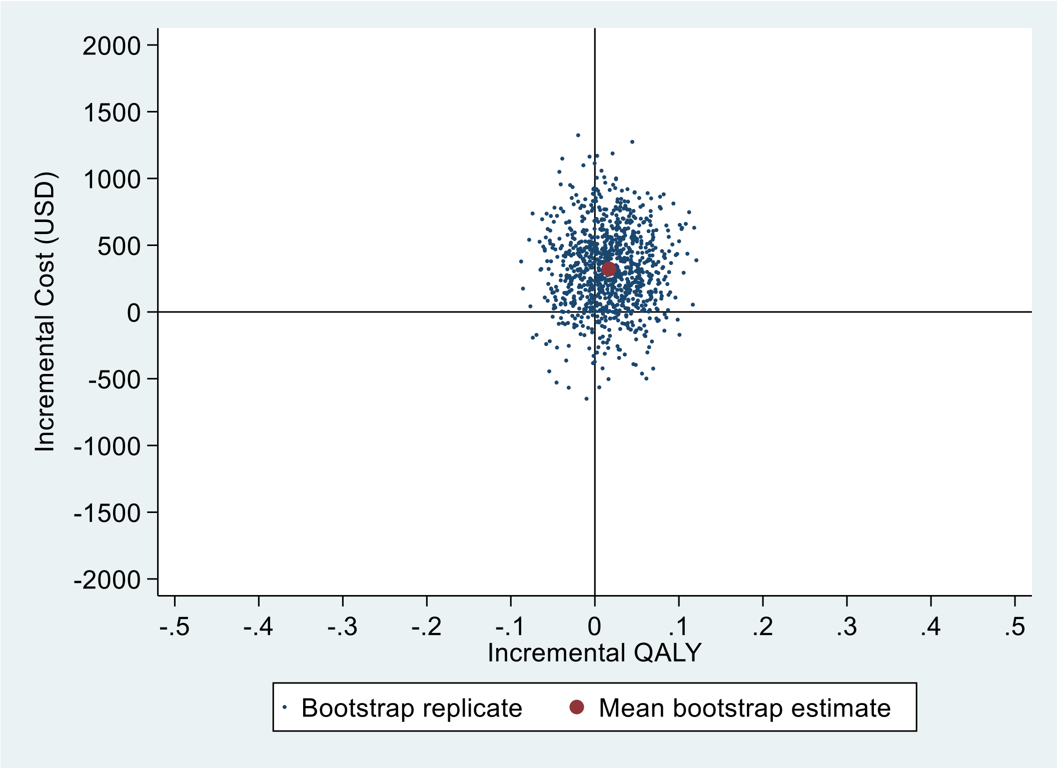

Supplement: Supplementary file 1 [file ijerph-20-03801-s001.zip › ijerph-2139323-supplementary.pdf]
